# 1000. Serotype 3 pneumococci evade activation of the classical complement pathway

**DOI:** 10.1093/ofid/ofab466.1194

**Published:** 2021-12-04

**Authors:** Rotem Lapidot, Mario Ramirez, Dayeun Lee, Ingrid L Scully, Bradford D Gessner, stephen pelton

**Affiliations:** 1 Boston University Medical Campus, Boston, MA; 2 Faculdade de Medicina, Universiudade de Lisboa, Lisboa, Lisboa, Portugal; 3 Boston Medical Center, Boston, Massachusetts; 4 Pfizer Vaccine Research and Development, Pearl River, New York; 5 Pfizer Vaccines, Collegeville, PA

## Abstract

**Background:**

Complement classical pathway (CCP) activation is the major mechanism leading to opsonophagocytic pneumococcal killing. Following immunization with 13-valent pneumococcal conjugate vaccine (PCV13), opsonophagocytic titers are lowest against serotype 3 among the 13 vaccine serotypes. Post licensure surveillance indicated early declines in serotype 3 invasive pneumococcal disease (IPD) were not sustained over time

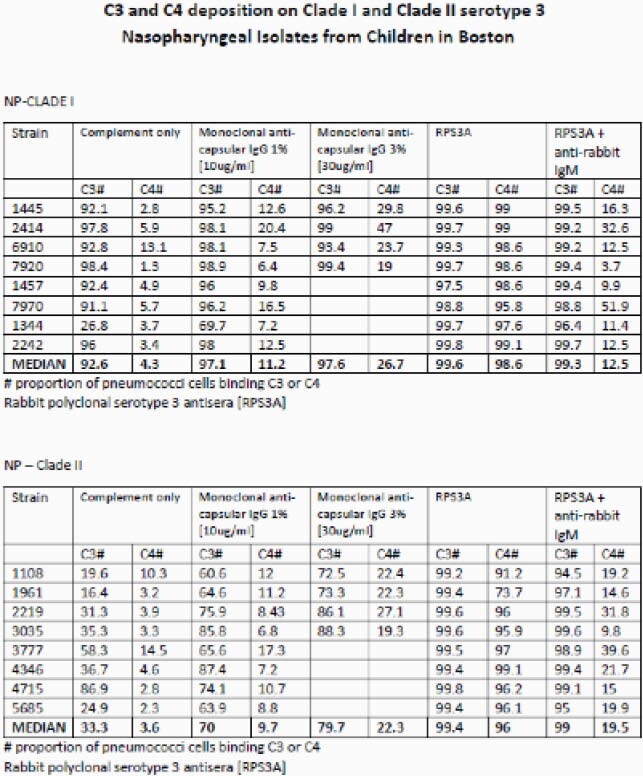

**Methods:**

Using flow cytometry, we measured C3 and C4 deposition on serotype 3 strains from children with IPD or nasopharyngeal [NP] carriage, and analyzed by clade. C4 deposition is an indicator of CCP, while C3 deposition is common to all complement pathways. We measured C3/C4 deposition on serotype 3 pneumococcal strains incubated with antibody depleted complement alone or with complement and the following antibodies: mouse monoclonal anti-capsular IgG or IgM, rabbit polyclonal serotype 3 antisera (IgG + IgM) [RPS3A] and RPS3A combined with anti-rabbit IgM, which blocks IgM function, leaving only polyclonal IgG

**Results:**

Serotype 3 strains demonstrated high variability in C3 binding when incubated with complement alone. RPS3A (containing both IgM+IgG) and monoclonal IgM activated CCP in all strains. Anti- serotype 3 monoclonal IgG and polyclonal IgG demonstrated absent or limited CCP activation; but activated alternative pathway in some strains. When analyzing complement deposition by clade, a lower proportion of clade II NP serotype 3 strains bound C3 when incubated with complement or monoclonal IgG, compared to clade Ia NP strains. Differences between clade Ia and II IPD strains were not apparent.

**Conclusion:**

Serotype 3 strains did not demonstrate activation of the CCP in the presence IgG and varied in C3 deposition. Pneumococcal strains that evade CCP activation may be less sensitive to opsonophagocytosis. Our findings suggest a mechanism by which serotype 3 carriage and disease may persist despite immunization with conjugate vaccine containing serotype 3 polysaccharide.

**Disclosures:**

**Rotem Lapidot, MD MSCI**, **Pfizer** (Consultant, Grant/Research Support, Advisor or Review Panel member) **Mario Ramirez, PhD**, **GlaxoSmithKline** (Advisor or Review Panel member)**Merck Sharp & Dohme** (Advisor or Review Panel member)**Pfizer** (Speaker’s Bureau) **Ingrid L. Scully, PhD**, **Pfizer** (Employee, Shareholder) **Bradford D. Gessner, MD, MPH**, **Pfizer Inc.** (Employee) **stephen pelton, MD**, **Merck Vaccines** (Advisor or Review Panel member, Research Grant or Support)**Pfizer, Inc.** (Consultant, Advisor or Review Panel member, Research Grant or Support)**Sanofi pasteur** (Advisor or Review Panel member, Research Grant or Support, DSMB)**Seqirus** (Consultant)

